# “Gaining or losing”: The importance of the perspective in primary care health services valuation

**DOI:** 10.1371/journal.pone.0188969

**Published:** 2017-12-05

**Authors:** Jesús Martín-Fernández, Gloria Ariza-Cardiel, Luz Mª Peña-Longobardo, Elena Polentinos-Castro, Juan Oliva-Moreno, Ana Isabel Gil-Lacruz, Héctor Medina-Palomino, Isabel del Cura-González

**Affiliations:** 1 Consultorio de Villamanta (CS Navalcarnero), Gerencia Asistencial de Atención Primaria, Madrid, Spain; 2 Facultad de Ciencias de la Salud, Universidad Rey Juan Carlos, Alcorcón, Spain; 3 Red de Investigación en Servicios Sanitarios y Enfermedades Crónicas (REDISSEC), Madrid, Spain; 4 Programa de Doctorado en Economía en la Universidad de Educación a Distancia (UNED), Madrid, Spain; 5 Unidad Docente Multiprofesional de Atención Familiar y Comunitaria Oeste, Gerencia Asistencial de Atención Primaria, Madrid, Spain; 6 Unidad de Apoyo a la Investigación, Gerencia Asistencial de Atención Primaria, Madrid, Spain; 7 Facultad de Ciencias Jurídicas y Sociales de Toledo, Universidad de Castilla La Mancha, Toledo, Spain; 8 Unidad Docente Multiprofesional de Atención Familiar y Comunitaria Norte, Gerencia Asistencial de Atención Primaria, Madrid, Spain; 9 Departamento de Dirección y Organización de Empresas, Escuela de Ingeniería y Arquitectura, Universidad de Zaragoza, Zaragoza, Spain; 10 Stichting Medina Care, Rotterdam, The Netherlands; Agencia de Salut Publica de Barcelona, SPAIN

## Abstract

**Rationale and objectives:**

Economic theory classifies an intervention as socially beneficial if the total Willingness to Pay (WTP) of those who gain exceeds the total Willingness to accept (WTA) of those who are harmed. This paper examines the differences in health system users’ valuation of a health care service in primary care setting based on the WTP and WTA perspectives, discussing the impact of personal and service variables, including risk attitudes, on these disparities.

**Method:**

Six hundred and sixty two subjects who asked for care in health centres in the Region of Madrid (Spain) were interviewed, using the contingent valuation method to estimate WTP and WTA. Patient sociodemographic characteristics, health needs, satisfaction with the service and risk attitude and behaviour under risk (measured by self-reported scales and lottery games respectively) were collected. Generalised Linear Models were used to estimate the association between the explanatory variables and the WTA/WTP ratio.

**Results:**

We obtained the WTA/WTP ratio for 570 subjects (mean 1.66 CI 95%: 1.53–1.79; median 1, interquartile range 1–2). People with higher education or in high social groups expressed WTA values closest to WTP. The opposite occurred in patients with the greatest health needs or who were born abroad. Self-reported expression of risk aversion appeared also related to increases in the WTA/WTP ratio. Satisfaction with the service evaluated was the most influential factor in the WTA/WTP ratio.

**Conclusion:**

Health need, difficulty in obtaining substitutes and satisfaction with the service could serve for profiling people averse to loss for health care services in primary care setting. Self-reported expression of risk aversion could also be related to increases in the WTA/WTP ratio. This would mean that these characteristics should be taken into account both in the design and implementation of new healthcare interventions, as in the making decision for disinvestment.

## Introduction

Understanding and disclosing the public’s preferences in order to shape health policy is an essential task that has been shown to improve the efficiency and quality of care [1]. Reproducibly measuring these preferences is a complex job, but there are methods for attributing value to goods or services for which there is no real market, as is the case with healthcare provided in the public setting. Contingent valuation (CV) is a method well grounded in economic theory which assumes that individual preferences can be interpreted in the form of a utility function, where two states (initial and final) can be compared in terms of the changes in the level of utility. This method has advantages in the case of the valuation of healthcare as it makes it possible to include use and non-use values, i.e. the value derived from viewing the product as a consumer good and the values relating to the very existence of the service [2].

The value attributed to a good or service with the CV method can be studied from the perspective of willingness to pay (WTP) or to accept (WTA). WTP is the maximum price a person would be prepared to pay to receive a service, while WTA is the minimum compensation the same person would require to abandon the service. In principle, the two approaches should yield similar values as they are valuations of the same good. However, numerous experiments have shown that the values obtained by WTA are consistently higher than those expressed by WTP when valuing the same good [3–8] irrespective of the method used to evaluate them [9].

Large positive differences between estimated WTA and WTP measures of the value of a gain, or the value of a loss, tend to accentuate the ambiguity that can arise under the rationale of the decision in health policy making. The Kaldor—Hicks compensation test classifies the policy intervention as socially beneficial if the total WTP of those who gain exceeds the total WTA of those who are harmed. A large difference between WTA and WTP will make this criterion more difficult to satisfy, hence favouring the status quo over the new policy intervention [8]. This may have implications for the design of policies and public funding or disinvesting services. Considering threshold values as determined by WTP for health gains or WTA for health losses relative to an origin on the cost-effectiveness plane, representing the endowment of health services for patients with current practice, they consequently suggest higher values on the cost-effectiveness plane, with losses in health relative to gains in health and a threshold line kinked at the origin, with greater slope (higher values), with negative incremental effects and costs, than with positive incremental cost and effects [10].

Economic theory attributes disparities in valuing a good or service using WTA and WTP to the fact that less substitutable goods are more difficult to replace and therefore greater compensation is required for their loss [11,12]. The scarcity of economic resources has a similar effect inasmuch as a person can reach the limit of their ability to pay before they are ready to accept compensation. Others argue that the disparity between WTA and WTP for the same good may well be related to hypothetical bias. The less information there is about the good valued (ex-ante studies) and the higher the costs of information, the greater the bias and the greater the WTA/WTP gap [13]. However, perhaps the idea that has been most explored when trying to understand these differences is risk aversion and, more recently, the endowment effect in which people who are given a good ask for a considerably higher price to relinquish it than similar people who do not have the good would be prepared to pay to buy it. New theoretical contributions from the field of psychology, such as the one provided by “prospect theory” (which is in the origin of the so called “behavioural economics”) emphasise aversion to loss as an explanation for the disparity between WTP and WTA, which means that the utility of the loss is greater than the utility of an equivalent gain [14,15].

In the above framework, valuation of certain health services based on WTA and WTP may be useful for distributing resources and more fully understanding service users’ representation of value, together with the perceived lack of substitutability or loss aversion with respect to the service. The purpose of this paper is to examine differences in health system users’ valuation of a service they receive based on the WTP and WTA perspectives by analysing the impact of the users’ risk attitude and other personal variables and service characteristics on the disparities between these two perspectives.

## Methods

### Design

Analysis of contingent valuation (CV) to estimate willingness to pay (WTP) or willingness to accept (WTA) for the healthcare received in a primary healthcare nursing visit in the public health system in the Community of Madrid (CM), one of the seventeen Autonomous Regions in Spain, with a population of 6.45 million people. This figure is higher than the populations of Denmark, Norway or Finland.

### Sample size and sample collection

Patients >18 years old patients, who had attended 1 of 23 health care centres in the Community of Madrid (Spain), asking for health care and who gave their written informed consent to participate in the project were interviewed in the context of a study of economic valuation of health care services [16]

The foreseen sample size (600 subjects) allowed building explanatory models including all the selected independent variables [17].

Inclusion criteria were being over 18, understanding Spanish, having experience with the purchase or sale of goods in order to understand the proposed scenarios, and giving written consent to be interviewed. Twenty three health centres in 6 of the 7 health divisions in the Community of Madrid were selected: 6 rural and 17 urban, 12 in high-income areas and 11 in low-income areas. The centres in each stratum were selected for convenience. The subjects were selected in each centre by systematic sampling of the appointments list.

### Measurement tools and variables included

Under the CV method individual interviews have to be conducted to estimate WTP and WTA. After the scenario had been presented, the question to determine WTP *“Imagine you have a similar health need to the one that brought you to the consultation today*, *and you must be attended by the same nurse who attended you today*, *but you have to pay for that service directly; how much would you be willing to pay for this consultation*?*”* In order to calculate WTA, the following question was used: *“[…] it was decided not to provide the service in the manner it has been provided until now [public health service*, *free access] and to compensate the citizen who will receive a check for the loss of the service*. *What would be the minimum quantity that you would require to receive in order not to feel harmed by the loss of this specific service*?*”*

The response to both questions was provided by a “double payment card” method (a system of payment cards in 2 phases) with an open upper limit, assuming that this would enable the user to behave as they would in an environment in which the same product was sold at different prices [18]. The first payment card only contained three values: less than €20, between €20 and €40 and more than €40. The second card was fitted to the valued expressed previously and contained the following values: €0, €5, €10, €15, €20, €25, €30, €35, €40, €45, €50, €55, €60, > €60. If the answer was “> €60”, the person was asked for an exact figure. The answer to the second payment card had to be consistent with the first answer. The values on the payment cards were chosen after a pilot survey in a group of 19 professionals and patients. This double payment-card method was used previously in order to minimize the range bias [19].

In all cases, the interview was performed at the time patients concluded the health consultation by the same interviewer, previously trained and knowledgeable in the method, at a location within the health centre but outside the health care area.

The main independent variable chosen was how people faced risks. This was evaluated in two ways: through the subjective perception of the interviewees (to measure the risk attitude), and through their responses to lottery games (to measure behaviour under risk), adapted from the German Socio-Economic Panel Study (SOEP) and others [20–22]. In the first case, the subjects were asked where they stood on a scale where 1 was the highest risk aversion and 10 the highest risk seeking. People were classified as “risk avoiding” when they offered a score below the median.

Subjects giving scores equal or under 5 on this scale were classified as “risk avoiding”.

In the second case, risk attitude was measured by two lottery games. In lottery 1 (L1), there was no possibility of loss and the maximum gain was €200. In the second lottery (L2), the prizes were the same, but the participant had to pay in €40 in order to take part, so the maximum loss was €40 and the maximum gain €200. The two lotteries were presented as theoretical games in which the subjects did not really win or lose money. When they chose not to play in any situation where the expected value was equal to or less than the certain outcome in one of the two lotteries, the subject was catalogued as “risk avoiding”.

The remaining variables were grouped into the following categories: health centre characteristics, patient demographic and social characteristics, health needs and use of services and satisfaction with the service.

The characteristics of the health centre were its rural or urban setting and the average disposable income of the area classified in the high and low tertile (2008 data, Statistics Institute. Community of Madrid).

Patient demographic characteristics were age, gender and nationality. Also noted were their level of education, “social class” based on 6 categories [23] and family income in thousands of euro adjusted by the number of people in the household using the method proposed by the OECD [24].

Health needs and use of services were measured by the existence of chronic conditions (ones that require continuous healthcare for more than 6 months), hospital admissions in the past year (including emergency stays lasting longer than 24 hours) and the perception of health-related quality of life (HRQoL) as measured by EQ-5D. The EQ-5D results were expressed on a visual scale and turned into utilities [25]. They were also asked about the existence of other insurance policies as a sign of experience in direct payment for health services.

A questionnaire validated in our environment was used to assess satisfaction with the service [26].

### Data analysis

The descriptive analysis is expressed in measures of central tendency and dispersion and by their 95% confidence intervals. We also used descriptions of the distribution if they were highly asymmetric.

We analysed the validity of the response by building an explanatory model in which the dependent variable was the WTA/WTP ratio.

Given the asymmetry in the distribution of the dependent variable (WTA/WTP ratio), we used Generalised Linear Models (GLM) to estimate the association between the explanatory variables and the WTA/WTP ratio [27]. The model in a general way can be expressed as follows:

$$\begin{array}{cc} g\left\{\left(y\right)\right\}=x_{ij}\beta_{j} & y\sim F \end{array}$$

where *y* is the dependent variable (WTA/WTP ratio), *g* {} is called the link function, F is the distributional family, *x*_ij_ is the covariables matrix and *β*_j_ is the coefficients vector.

“Identity function” was selected as link function *g* {}, defined as

g{E(y)}=E(y)

The selected distributional family was gamma family since the Akaike Information Criterion (AIC), makes it possible to identify that this was the distribution which best fits the dependent variable to be analysed. The GLM models were estimated by maximum likelihood methods. This enabled us to obtain predictions directly without any logarithmic transformation, which take into account heteroscedasticity and provide consistent estimates [28]. In addition, since there might have been some association between the reported WTP/WTA ratio and the health centre to which the individuals belong, we decided to adjust the models by clusters.

Given the possible correlation between educational level and socioeconomic status, as well as the possible correlation between risk attitudes (measured by the self-reported score) and behaviours under risk (measured by lotteries), we decided to make 4 different models: model 1 including education level and risk seeking by lotteries; model 2 including socioeconomic status and risk seeking by lotteries; model 3 including educational level and self-reported risk seeking; and model 4 including socioeconomic status and self-reported risk seeking.

The models were selected out of all the possible options due to their fit with our theoretical model, their ability to adjust our observations and the principle of parsimony.

### Ethical and legal issues

The study was reviewed and approved by the Alcorcón Hospital Foundation’s Ethics Committee (Institutional Review Board) on June 25^th^ 2009. The entire research process was guided by the ethical principles contained in the Declaration of Helsinki (revised Seoul 2008). All individuals in the study were asked for their written consent to take part in it and the data were stored and processed anonymously, thus meeting the requirements of national legislation.

## Results

A total of 662 subjects were included. Subjects who refused to participate (n = 95) did not differ in age and gender from those included. There were no differences in refusals in centres of high or low income areas.

Eighty one of the subjects included (12.2%) expressed a WTP of €0. When asked about their WTA, 14 people (2.1%) did not answer and 13 subjects (2.0%) said 0. Just 2 of the subjects who expressed a WTP greater than 0 did not answer the question about WTA and another 2 gave an answer of 0. The median value expressed for WTP was €10 (interquartile range: €5-€20), and for WTA €20 (interquartile range: €10- €30). We obtained the WTA/WTP ratio for 570 subjects with a mean of 1.66 (CI 95%: 1.53–1.79). The median distribution of the WTA/WTP ratio was 1 (interquartile range: 1–2), and 193 of the 570 subjects had a WTA/WTP ratio above 1 (33.9%, CI 95%: 30.0–37.8%).

Table 1 shows the characteristics studied for subjects where we were able to estimate the WTA/WTP ratio. There were no significant differences in any of the variables studied with respect to all the subjects included in the study.

**Table 1:** Characteristics of the people who expressed WTA and WTP.

	Mean (CI 95%)	Median (CI range)	Percentages (CI 95%)
Age in years	65.0 (63.6–66.4)	69 (54–78)	
Gender (female)			60.0 (55.9–64.0)
Spanish			94.7 (92.9–96.6)
Chronic illnesses			81.4 (78.2–84.7)
Hospital admissions in the last year			27.9 (24.2–31.7)
Overall satisfaction (1 the worst—5 the best)	4.89 (4.86–4.93)	5.0 (5.0–5.0)	
EQ-5D-VAS	67.0 (65.2–68.8)	70 (50–80)	
EQ-5-D utilities	0.70 (0.68–0.72)	0.78 (0.52–1.00)	
Other insurance			16.7 (13.6–19.8)
Educational level			
Illiterate and no schooling			27.4 (23.7–31.0)
Primary education			33.9 (30.0–37.8)
Secondary education			23.5 (20.0–27.5)
Higher education			15.3 (12.3–18.2)
Social class			
Manager, Director			10.0 (7.5–12.5)
Intermediate positions			13.9 (11.0–16.7)
Skilled non-manual worker			26.8 (23.2–30.5)
Skilled manual worker			23.0 (19.5–26.4)
Partially skilled manual worker			11.1 (8.5–13.6)
Unskilled manual worker			15.3 (12.3–18.2)
Adjusted family income (thousands of €)		0.750 (0.600–1.050)	
Risk avoiding (self-perception scale)			57.4 (53.6–61.2)
Risk avoiding (lotteries)			59.1 (55.3–62.9)

WTA: Willingness to Accept; WTP: Willingness to Pay.

CI 95%: Confidence interval 95%; CI range, interquartile range (25th-75th percentile).

Table 2 shows the best equation to explain the WTA/WTP ratio when risk attitude was measured by means of the self-reported score. Expressed risk avoiding attitude was related to increases in the WTA/WTP ratio of 16% to 18% (models 1 and 2).

**Table 2:** Factors explaining the WTA/WTP ratio, measuring self-reported risk score.

	Model 1: Educational level adjustment variable		Model 2: Social group adjustment variable	
Variable	Coefficient (CI 95%)	P value	Coefficient (CI 95%)	P value
Spanish vs. foreign	-0.74 (-1.75- -0.04)	0.036	-0.65 (-1.52–0.23)	0.148
HRQoL (utilities)	-0.25 (-0.53–0.02)	0.073	-0.27 (-0.51- -0.04)	0.020
Satisfaction (scale 1–5)	0.25 (0.16–0.33)	< 0.001	0.28 (0.18–0.38)	< 0.001
No schooling vs. primary education	0.39 (0.12–0.67)	0.006	-	
Secondary vs. primary education	-0.06 (-0.42–0.30)	0.740	-	
Higher vs. primary education	-0.32 (-0.56- -0.08)	0.009		
Intermediate positions vs. manager or directors	-		0.40 (0.08–0.73)	0.015
Skilled non-manual worker vs. manager or directors	-		0.33 (0.07–0.65)	0.045
Skilled manual worker vs. manager or directors	-		0.47 (0.09–0.86)	0.016
Partially skilled manual worker vs. manager or directors	-		0.37 (0.09–0.63)	0.009
Unskilled manual worker vs. manager or directors	-		0.91 (0.58–1.25)	< 0.001
Self-reported risk avoiding (under the median)	0.16 (0.00–0.33)	0.049	0.18 (0.01–0.35)	0.044
Model properties (adjusted for age and gender)	N = 569 Akaike Information criterion = 3.004		N = 569 Akaike Information criterion = 3.008	

Table 3 shows the model adjusted by the behaviour under risk (lottery games). Risk avoiding behaviour, measured by lotteries, was not associated with the ratio between WTP and WTA expressed for lotteries (models 3 and 4).

**Table 3:** Factors explaining the WTA/WTP ratio, measuring risk aversion by lotteries.

	Model 3: Educational level adjustment variable		Model 4: Social group adjustment variable	
Variable	Coefficient (CI 95%)	P value	Coefficient (CI 95%)	P value
Spanish vs. foreign	-0.74 (-1.48–0.01)	0.048	-0.62 (-1.50–0.26)	0.165
HRQoL (utilities)	-0.27 (-0.59–0.04)	0.084	-0.32 (-0.56- -0.05)	0.017
Satisfaction (scale 1–5)	0.24 (0.14–0.33)	<0.001	0.27 (0.17–0.38)	< 0.001
No schooling vs. primary education	0.40 (0.12–0.68)	0.005	-	
Secondary vs. primary education	-0.07 (-0.45–0.31)	0.712	-	
Higher vs. primary education	-0.33 (-0.58–0.07)	0.011	-	
Intermediate positions vs. manager or directors	-		0.43 (0.08–0.79)	0.017
Skilled non-manual worker vs. manager or directors	-		0.36 (0.05–0.67)	0.022
Skilled manual worker vs. manager or directors	-		0.48 (0.12–0.84)	0.010
Partially skilled manual worker vs. manager or directors	-		0.40 (0.14–0.66)	0.003
Unskilled manual worker vs. manager or directors	-		0.94 (0.618–1.27)	< 0.001
Risk averse in lotteries	-0.06 (-0.29–0.16)	0.569	-0.07 (-0.27–0.13)	0.479
Model properties (adjusted for age and gender)	N = 566. Akaike Information criterion = 3.002		N = 566. Akaike Information criterion = 3.006	

For lower levels of education (illiterate and no schooling), the WTA/WTP ratio increased by about 39–40% compared to primary education, while for people with higher education this ratio decreased by similar range (32–33%, models 1 and 3). When adjusted for social class, the most disadvantaged classes increased their expressed WTA/WTP ratio up to 90% compared to the better-off social groups (models 2 and 4). The nationality of the subject was associated with the WTA/WTP ratio, which was about 74% lower for Spaniards than for foreigners in models 1 and 3. This association lost statistical significance after adjusting for social group. Satisfaction with the service evaluated was consistently associated with the WTA/WTP ratio. The difference between the ends of the satisfaction scale (4 points) expressed mean differences in the WTA/WTP ratio of between 96% and 112% (models 1, 2, 3 and 4). HRQoL expressed in utilities was also significantly associated with the differences between expressed WTP and WTA. Patients who perceived better HRQoL expressed lower WTA/WTP ratios, with about 30% difference between the ends of the HRQoL measurement scale (models 2 and 4). Presenting chronic diseases, having been admitted to hospital and having taken out other insurance did not contribute explanatory power to the disparities in the WTA/WTP ratio. Family income showed collinearity with social group and educational level, so it was not included in the models.

Health centre characteristics were not related to the WTA/WTP ratio.

Models were repeated by attributing to interviewees who had offered zero WTP, the same WTP values of people of the same age and gender living in neighbourhoods with similar income and belonging to the same social group. Neither the explanatory factors nor the strength of the associations changed significantly.

## Discussion

The perception of a health service such as the one evaluated here is different from the WTP and WTA perspective, even when this valuation is conducted with the same subjects and at the same time. Expressed WTA was greater than WTP by a mean 66% for the same service, although over half of the interviewees offered the same value for both questions. In addition to factors related to disparities between WTP and WTA which had been studied quite widely, such as socioeconomic status, age and satisfaction with the product, it seems that the attitude to risk may be associated with differences in the expression of these values.

The disparities found between WTA and WTP for the service studied are consistent with the literature. The reason for this disparities are usually grouped in economics, into three types of categories: those related to the elicitation technique [29], those related to the nature of the good studied [30] and those intrinsic to the subject who makes the valuation, where we would highlight risk avoiding [14]. Although some explanations of the WTP-WTA divergence operates within the welfare economics theory, others challenge this framework [31].

As for the type of technique, it seemed that WTA/WTP disparities were smaller with iterative deal questions than with payment card designs and also when the question was put to the same subjects [4] as in this case.

It had been hypothesised that the disparities between WTA and WTP were higher in services that could not be found in ordinary markets or, in the case of public goods, than when the experiences related to goods that were easy to find in the market [30]. Health care is a private good, in the economic sense, but it owns some "public good" characteristics, as its potential positive externalities (or negative if the need is not attended). In the field of health, the WTA/WTP ratio varies depending on the nature of the evaluated service, reaching a value of 4 when surgical techniques to improve hearing for children were assessed [5], and approaching 1 where the provision of informal care to the chronically ill was evaluated [32]. Also related to the type of good studied are the ability to find substitute goods and the cost of obtaining information about these exchanges, both of which are characteristics that might increase the WTA/WTP ratio [13]. In our case we did not find that subjects who used substitute goods (other insurance) expressed a lower WTA/WTP ratio, although it did happen with Spanish subjects compared to foreigners. Better understanding of the environment’s healthcare services can be attributed to subjects born in Spain as a group due to more prolonged contact with them.

There are two other features related to the good evaluated and which may influence the WTA/WTP ratio: the need for this good and satisfaction with its use. Those subjects with worse health expressed greater differences in the WTA/WTP ratio, as expected However, the factor with the most individual impact on this ratio was satisfaction. This may be related to a perceived lack of substitutability of the service or uncertainty about substitute goods, factors that we found widen the differences in the valuations from the different perspectives [12,33]

A third category of explanatory reasons for the disparities between WTA and WTP concern the subject who values the good, playing the role of demand. The literature suggested there was a greater gap between WTA and WTP in older people and in women [6,15,34]. However, although age and gender were introduced as an adjustment factor to enhance the explanatory power of the model, they were not statistically significant. There are other individual characteristics which welfare theory argues play a fundamental role in explaining the differences found in the valuation of a good or service by WTA and WTP. Perhaps the most researched is the income effect, by which an individual may not have sufficient resources to acquire a utility but does demand compensation for losing it. In our case, family income was not included in the best model, even though it was an explanatory variable for disparities between WTA and WTP, as there was clear collinearity with social group and educational level which meant not all of them could be in the model. Both belonging to a “high” social group and having a higher educational level point to the most advantaged social sectors, and both characteristics were associated with a lower WTA/WTP ratio. Some authors suggest that the differences between WTA and WTP are often too large for the income effect to be the only variable that explains them [35]; in this case we found that the characteristics indicating an individual’s socioeconomic status were significant in explaining the different valuations but were not fundamental.

The subject characteristics explaining the difference in WTP and WTA that have little to do with classical economic theory, and are closer to recent theoretical developments in psychology, are those that focus on resistance to loss or risk aversion and may vary for each good evaluated. Risk aversion has been found to be a variable that explains behaviour both in risk environments and in decisions which in principle do not involve risk [15]. This paper makes two new contributions in this respect. Firstly, it shows that the more “risk avoiding” profiles are those with a higher WTA/WTP ratio, i.e. the different valuations for gains and losses may be associated with the valuation of the good assessed, and it includes this fact in the specific area of a health-related service. Secondly, it points out that the way in which risk attitude is disclosed is not neutral, since results and conclusions may vary depending on the disclosure method selected. Risk aversion as an attitude was related to the WTP/WTA gap, but behaviour under risk was not. This could be due to a methodological issues but it is known that, attitudes and behaviors are correlated but not always aligned [36,37].

This paper may have some limitations. We cannot rule out the possibility of strategic bias, which could lead to much higher figures for WTA compared to WTP because the subject believes that in this way they can express a reluctance to change the service. This is particularly important given that when the study was conducted there was an open debate about whether or not to change health service copayment schemes.

Other type or limitation is related to the difficulty in assessing the significance of the rate of zero answers to the WTP question. In this case it was not higher than that found in other studies evaluating health services [19,38], and it does not appear to have any influence on the explanatory variables for the WTA/WTP ratio since the model with values “attributed” to the zeros had similar characteristics to the one presented. What may be a surprise is the high percentage of responses in which WTA is identical to WTP. We cannot be sure about the effect the design may have had on the results presented. Open-ended payment cards were used to learn WTA and WTP values, we referred to out-of-pocket payments and the same subjects were asked about the two values for the same service. The former tends to overestimate the WTA/WTP ratio, while the latter two do the reverse [4].

Finally, behaviours under risk were disclosed using hypothetical lotteries and not real lotteries with possible monetary gains and losses for the participants.

There are also several strengths in the design that could be underlined. Given that the description of the scenario may have implications for the expressed values [39], we chose as realistic a description as possible, making reference to a good immediately obtained. The payment card format shows advantages and inconveniences compared with other methods of eliciting values for WTP and WTA [40], but it is a commonly accepted tool. To minimize any possible bias that the range presented in the card might introduce, we asked a question in two phases, the first with a wide range and the second attempting to determine the real value stated. As recommended in the principal guides, the survey was a face to face interview, managed by a trained personnel [41], outside the health care process, immediately after the visit.

Although the present results cannot be extrapolated to other services received for other people in other contexts (i.e., other health services organizations or other cultural environments), it would appear that the disparities between WTA and WTP when evaluating a health-related service are consistent, and some of the assumptions drawn from outside classical economic theory need to be used in order to understand them. It is well known and documented that subjects behave differently with respect to gains compared to losses [42]. However, this point is not only relevant to learning what the value attributed to a particular service is, but in itself may also be an expression of the rejection of its loss. A debate exists about the opportunity to include loss aversion in health policy planning, since it affects resource allocation not only through the cost-effectiveness ratio but also through societal values concerning the distribution of health care resources [43]. It may even be that the preference for a certain good (or service) depends on whether it is going to be introduced or withdrawn, on when this will take place and on the quantity of wellbeing that it engenders [44]. This factor should be borne in mind when disinvestment approaches concerning clinical services and public health interventions should being planned [45].

Individual preferences should be aggregated in any way to shape social preferences. One traditional debate in the economic field is whether a Social Welfare Function (SWF), could be obtained. This function can be understood as a set of individual preferences on possible social states and associates a social preference to each possible configuration of individual preferences, and can then be used to rank economically feasible allocations of resources in terms of the social welfare they entail. The SWF should fulfil a list of logical norms in the process of aggregation of preferences (completeness, reflexivity, transitivity, universality or unrestricted domain condition, unanimity, Pareto efficiency and independence of irrelevant alternatives). As early as 1950, Kenneth J. Arrow demonstrated that this may be incompatible with democratic norms [46], hence some of the above conditions must be relaxed (i.e. universality or unrestricted domain status, unanimity or Pareto criterion). The discussion about how to aggregate individual preferences in a SWF, which will be the main tool for decision-making, continues today [2].

Cost-benefit analysis has been proposed as a valid method for analyzing changes in a SWF. While the SWF methodology relies on an interpersonally comparable utility function, CBA measures impacts on individuals in terms of money (individual willingness-to-pay or accept). A given outcome (a possible consequence of policy choice) is converted into a “vector,” of utility numbers, one for each person in the population. These vectors are then ranked by some rule—most simply a utilitarian function. Utilitarian function focuses on the maximisation of benefits, adding together the benefits to each individual regardless of their characteristics. If income and other determinants of individual utility are symmetrically distributed among the winners and losers of a given policy choice, CBA is a reasonable framework for a utilitarian SWF [47]. However, the WTA-WTP differences for the same service accentuate an existing problem. When a (health) policy is going to be developed, the fundamental problem is the need to compare benefits provided to some people with harms imposed on others [8]. Maybe it is more useful for policy makers to address the fundamental question, whether the benefits to those who gain from the policy justify the harms to those who lose, but keeping in mind that perceived utility is different under gain or losses perspective and that there is a profile of people who express greater differences between two perspectives.

## Conclusion

The valuation of primary care health services varies from the gain or loss perspective assessed by WTP and WTA respectively. Both the need for and the difficulty in obtaining substitute goods, or satisfaction with the service, increase the gap between the two valuations as had been suggested previously. The income effect may also play a role in the disparity between WTA and WTP. Expressed risk avoiding could be associated with an increase in the WTA/WTP ratio, which is consistent with psychological theories that propose loss aversion as a key element in explaining this fact. Joint analysis of the factors that form part of the subject’s personality, along with other socioeconomic characteristics and factors that define the quality of the good or service, need to be built into analysis of the valuation of goods and services in healthcare.

So a profile of loss aversion to health care services could be identified and this would mean that these characteristics should be taken into account both in the design and implementation of new healthcare interventions services, as in the making decision for disinvestment.

## Supporting information

S1 Dataset S1 Dataset.xls. (XLS)

S1 Questionnaire S1 Questionnaire English version.doc. (DOC)

S2 Questionnaire S2 Questionnaire Spanish version.doc. (DOC)
