# Supplementary material for: “Gaining or losing”: The importance of the perspective in primary care health services valuation
Source: PLoS One. 2017 Dec 5;12(12):e0188969. doi: 10.1371/journal.pone.0188969 (PMC5716530; doi:10.1371/journal.pone.0188969)
Supplement: S1 Questionnaire — (DOC) [file pone.0188969.s002.doc]

This questionnaire was administered as part of the study "Economic value perceived by the user of nurse consultations in primary care setting", between October 2011 and January 2012.

**Section 0 (filled by the nurse)**

**Your age is ___years**

** Man  Woman**

**In the last year, has the patient been admitted to a hospital? (including >24 hours stay in the emergency room)**

** Yes  No**

**Number of visits to the nurse in the last year**: __times.

**Number of visits to the family physician in the last year**: __times.

**The patient has a chronic pathology:**

**** Yes **** No

**Total number of chronic pathologies:** ___

**Has the patient been diagnosed as a smoker? ** Yes **** No

**Has the patient been diagnosed as a former smoker? ** Yes **** No

**Has the patient been diagnosed as at risk-drinker? ** Yes **** No

**Has the patient been diagnosed as alcohol-depending? ** Yes **** No

**Has the patient stated consumption of other drugs? ** Yes **** No

**Section 1**

**We are going to ask you for information about yourself and your state of health**

**You are from …?**

**** Spain

- The rest of the European Union, including Iceland, Norway and Switzerland.
- Latin America

****  North Africa

- Sub-Saharan Africa
- Other non-EU European countries

Includes: Turkey, Croatia, Macedonia, Albania, Armenia, Azerbaijan, Byelorussia, Bosnia, Georgia, Liechtenstein, Moldavia, Montenegro, Russia, Serbia, and Ukraine.

- Asia
- Other…

**If you were not born in Spain, during how many years have you been living in Spain? ___ years**

**Do you have additional health insurance?**

**** Yes **** No

**Which one?**

**** Asisa

- Sanitas

**** Adeslas

- Other (specify):

**If you do have additional health insurance, who pays for it?**

**** Your company **** Yourself

**By placing a checkmark in one box in each group below, please indicate which statements best describe your state of health today.**

Mobility:

**** I have no problems walking

**** I have some problems walking

**** I have to stay in bed

Personal care:

**** I have no problems with personal care

**** I have some problems to wash and dress myself

**** I cannot wash or dress myself

Daily activities (e. g., work, study, housework, family activities or during leisure time):

**** I have no problems doing my daily activities

**** I have some problems doing my daily activities

**** I cannot perform my daily activities

Pain/discomfort:

**** I have no pain or discomfort

- I have moderate pain or discomfort
- I have significant pain or discomfort

Anxiety/depression:

**** I am not anxious or depressed

**** I am moderately anxious or depressed

**** I am very anxious or depressed

Compared with my general state of health during the last 12 months, my state of health today is:

**** Better

**** Same

**** Worse

To help people describe how good or bad their state of health is we have drawn a scale similar to a thermometer on which 100 marks the best state of health imagined and 0 the worst state of health imagined. We would like you to indicate on this scale, in your opinion, how good or bad your state of health is *today*.

Better

State

of Health

Worst

State

of Health

Your health status today

**Section 2**

**Scenerio 1.**

“Imagine you have a similar health need to the one that brought you to the consultation today, and you must be attended by the same nurse who attended you today, but you have to pay for that service directly; how much would you be willing to pay for this

consultation?

Card 1. How much money would you be willing to pay for this consultation?

** ** A: 0-20 euros

- B: 21-40 euros
- C: >40 euros

Card 2. How much money would you be willing to pay for this consultation?

** ** A: 0 euros

- B: 5 euros
- C: 10euros
- D: 15euros
- E: 20euros

- F: 25euros
- G: 30euros
- H: 35euros
- I: 40euros
- J: 45euros
- K: 50euros
- L: 55euros
- M: 60euros
- N:>60euro**s**

**If you have checked option A, it was due to:**

- I cannot afford to pay for this service
- I am not willing to pay for this service
- I do not find relevant this question
- Other reasons

**If you have checked option N, I would be willing to pay ___ euros**

**Scenario 2.**

In this new situation imagine that it was decided not to provide the service in the manner it has been provided until now [public health service, free access] and to compensate the citizen who will receive a check for the loss of the service.

Card 1

What would be the minimum quantity that you would require to receive in order not to feel harmed by the loss of this specific service?”

** ** A: 0-20 euros

- B: 21-40 euros
- C: >40 euros

Card 2

What would be the minimum quantity that you would require to receive in order not to feel harmed by the loss of this specific service?”

** ** A: 0 euros

- B: 5 euros
- C: 10euros
- D: 15euros
- E: 20euros
- F: 25euros
- G: 30euros
- H: 35euros
- I: 40euros
- J: 45euros
- K: 50euros
- L: 55euros
- M: 60euros
- N:>60euros

**If you have checked option N, you would receive, at least ___ euros**

**Section 3**

**Regarding the relationship with your nurse, several phrases about what a person can feel about it will be presented. Choose the appropriateness of each sentence with respect to your case by marking one number by sentence (remember that what you say is confidential and no one will be able to access your answers).**

**1. My nurse listens to me when I explain my health problems**

| 1  Strongly disagree | 2  Disagree | 3  Neutral | 4  Agree | 5  Strongly agree |
| --- | --- | --- | --- | --- |

**2. My nurse is kind and treat me courteously**

| 1  Strongly disagree | 2  Disagree | 3  Neutral | 4  Agree | 5  Strongly agree |
| --- | --- | --- | --- | --- |

**3. I trust my nurse, I believe what she says**

| 1  Strongly disagree | 2  Disagree | 3  Neutral | 4  Agree | 5  Strongly agree |
| --- | --- | --- | --- | --- |

**4. I am totally satisfied with the consultation to this nurse**

| 1  Strongly disagree | 2  Disagree | 3  Neutral | 4  Agree | 5  Strongly agree |
| --- | --- | --- | --- | --- |

**5. The nurse has paid close attention to examining all the problems**.

| 1  Strongly disagree | 2  Disagree | 3  Neutral | 4  Agree | 5  Strongly agree |
| --- | --- | --- | --- | --- |

**6. I will follow the advice of the nurse because I think they are very appropriate**.

| 1  Strongly disagree | 2  Disagree | 3  Neutral | 4  Agree | 5  Strongly agree |
| --- | --- | --- | --- | --- |

**7. I felt comfortable talking to the nurse about very personal issues**.

| 1  Strongly disagree | 2  Disagree | 3  Neutral | 4  Agree | 5  Strongly agree |
| --- | --- | --- | --- | --- |

**8. The time I spent with the nurse has been quite a bit short.**

| 1  Strongly disagree | 2  Disagree | 3  Neutral | 4  Agree | 5  Strongly agree |
| --- | --- | --- | --- | --- |

**9. The nurse has given me full information about my treatment**.

| 1  Strongly disagree | 2  Disagree | 3  Neutral | 4  Agree | 5  Strongly agree |
| --- | --- | --- | --- | --- |

**10. Some aspects of the nurse consultation might have been better**.

| 1  Strongly disagree | 2  Disagree | 3  Neutral | 4  Agree | 5  Strongly agree |
| --- | --- | --- | --- | --- |

**11. There are some things the nurse does not know about me.**

| 1  Strongly disagree | 2  Disagree | 3  Neutral | 4  Agree | 5  Strongly agree |
| --- | --- | --- | --- | --- |

**12. The nurse has listened very carefully to everything I have said.**

| 1  Strongly disagree | 2  Disagree | 3  Neutral | 4  Agree | 5  Strongly agree |
| --- | --- | --- | --- | --- |

**13. I think the nurse has treated me in a personalized way.**

| 1  Strongly disagree | 2  Disagree | 3  Neutral | 4  Agree | 5  Strongly agree |
| --- | --- | --- | --- | --- |

**14. The time I've been with the nurse has not been enough to tell her everything I wanted**.

| 1  Strongly disagree | 2  Disagree | 3  Neutral | 4  Agree | 5  Strongly agree |
| --- | --- | --- | --- | --- |

**15. After the consultation with the nurse I understand my health problem much better.**

| 1  Strongly disagree | 2  Disagree | 3  Neutral | 4  Agree | 5  Strongly agree |
| --- | --- | --- | --- | --- |

**16. The nurse has been interested in me not only because of my illness, but also as a person.**

| 1  Strongly disagree | 2  Disagree | 3  Neutral | 4  Agree | 5  Strongly agree |
| --- | --- | --- | --- | --- |

**17. The nurse knows everything about me.**

| 1  Strongly disagree | 2  Disagree | 3  Neutral | 4  Agree | 5  Strongly agree |
| --- | --- | --- | --- | --- |

**18. I think the nurse really knew what I was thinking**.

| 1  Strongly disagree | 2  Disagree | 3  Neutral | 4  Agree | 5  Strongly agree |
| --- | --- | --- | --- | --- |

**19. I would have liked to have spent more time with the nurse.**

| 1  Strongly disagree | 2  Disagree | 3  Neutral | 4  Agree | 5  Strongly agree |
| --- | --- | --- | --- | --- |

**20. I am not entirely satisfied with the visit to the nurse**

| 1  Strongly disagree | 2  Disagree | 3  Neutral | 4  Agree | 5  Strongly agree |
| --- | --- | --- | --- | --- |

**21. It would be difficult for me to talk to the nurse about personal issues.**

| 1  Strongly disagree | 2  Disagree | 3  Neutral | 4  Agree | 5  Strongly agree |
| --- | --- | --- | --- | --- |

**Section 4**

**Next, we are going to ask you a series of questions about your acceptance or rejection of risk. There is no right or wrong answer, and we would only like to know your attitude towards certain situations.**

**Would you say you're risk-averse or risk-prone** **when there is a possible benefit? Please indicate on the scale from 1 to 10, how much of a risk-taker do you consider yourself?**

**Totally risk-averse = 1**

**Totally risk-prone = 10**

| **1** | **2** | **3** | **4** | **5** | **6** | **7** | **8** | **9** | **10** |
| --- | --- | --- | --- | --- | --- | --- | --- | --- | --- |

**Now imagine that you can take part in a lottery game in which you can choose between one of two boxes. But first, the quiz director offers you increasing amounts of money if you stop playing . One box contains €200 and the other one is empty. If you choose to compete, your prize will be the contents of the box you choose.**

**Mark your preferences in each situation:**

** They offer you €40 versus One of the two boxes **

** They offer you €70 versus One of the two boxes **

** They offer you €100 versus One of the two boxes **

** They offer you €130 versus One of the two boxes **

** No response**

**Imagine now that the lottery game rules have changed. The quiz director will also offer you increasing amounts of money each time. But, if you choose to compete you must first pay a fixed amount of €40. The prize for competing is the same as above, the contents of the box you choose, knowing that one contains €200 and the other one is empty.**

**Mark your preferences in each situation:**

** They offer you €0 versus One of the two boxes **

** They offer you €30 versus One of the two boxes **

** They offer you €60 versus One of the two boxes **

** They offer you €90 versus One of the two boxes **

** No response**

**Section 5**

**Finally, we are going to ask you for some statistical information that will help us to classify your answers and to interpret the results of the study:**

**The number of people who live in my home is: ____ persons**

**The number of them who are sixteen or older _____ .**

**The number of them who are fifteen or younger _____.**

**Your main occupation at present is:**

**** Housewife.

**** Student.

**** Worker.

**** Unemployed.

- Retired.

**The highest level of education you have completed is:**

**** I do not know how to read or write.

**** No education.

**** Primary studies.

- Secondary studies.
- Superior studies.

**My last paying job was:**

**** I Manager, director, Higher professional occupations.

**** II Mid-level position or sales manager.

**** III Non-manual skilled worker.

**** IVa Skilled manual worker.

**** IVb Partially-skilled manual worker.

**** V Non-skilled manual worker.

In the case of not having a paid job, the member with the highest career category within the family unit is considered. Unemployed, temporary or permanent incapacitated, or retired are classified according to the last job held.

**The monthly income of your family unit (adding up all family members who live in your home) is in the range indicated with the letter:**

**** A: Less than 600 euros

- B: 600- 1200 euros.
- C: 1200-1800 euros.
- D: 1800-2400 euros.
- E: 2400- 3600 euros.
- F: 3600- 4800 euros.
- G. 4800-6000 euros.
- H: 6000-7200 euros.
- I: More than 7200 euros.
