# Supplementary material for: “Gaining or losing”: The importance of the perspective in primary care health services valuation
Source: PLoS One. 2017 Dec 5;12(12):e0188969. doi: 10.1371/journal.pone.0188969 (PMC5716530; doi:10.1371/journal.pone.0188969)
Supplement: S2 Questionnaire — (DOC) [file pone.0188969.s003.doc]

Este cuestionario se administró como parte de la encuesta para el Estudio sobre la percepción de valor económico del usuario de las consultas de enfermería en Atención Primaria entre los meses de octubre de 2011 y enero de 2012.

**Apartado 0 (a cumplimentar por la enfermera)**

**Su edad es de ___años**

** Varón  Mujer**

**En el último año Ud ¿el paciente ha sido ingresado en el Hospital?**  (incluido permanecer más de 24 horas en Urgencias)

**** Sí **** No

**Número de consultas a la enfermera en el último año** : __veces.

**Número de consultas del médico de familia en el último año** : __veces.

**Número total de patologías crónicas** (Cualquier problema de salud que requiera atención continuada por periodos superiores a 6 meses): ____

**¿Tiene el paciente diagnóstico en la historia de fumador? ** Sí **** No

**¿Tiene el paciente diagnóstico en la historia de ex-fumador? ** Sí **** No

**¿Tiene el paciente diagnóstico de bebedor excesivo de alcohol?** Sí **** No

**¿Tiene el paciente diagnóstico de bebedor de riesgo? ** Sí **** No

**¿Tiene el paciente diagnóstico de consumo de otros tóxicos? ** Sí **** No

**Apartado I**

**Vamos a preguntarle algunos datos relativos a usted y a la consulta que acaba de realizar**

**¿Ud. es natural de…?**

**** España

- Resto de Unión Europea más Islandia, Noruega y Suiza.
- Latinoamérica

**** Magreb

- África Subsahariana
- Otros países Europeos no UE

Incluye miembros candidatos (Turquía , Croacia y Macedonia) y Albania, Armenia, Azerbaiyán, Bielorrusia, Bosnia, Georgia, Kazajistán, Lietchtenstein, Moldavia, Montenegro, Rusia, Serbia y Ucrania.

- Asia
- Otros:…

**Si no ha nacido en España, lleva usted viviendo en España: ______años**

**¿Tiene Ud otro sistema de aseguramiento?**

**** Sí **** No

**¿Cuál?**

**** Asisa

- Sanitas

**** Adeslas

- Otra (especificar): _____________________________

**En caso de tener otro sistema de aseguramiento, la prima la paga**

**** Su empresa **** Ud. mismo

**Respecto a su estado de salud en el día de hoy, señale la respuesta de cada apartado que mejor lo describa :**

Movilidad

**** No tengo problemas para caminar

**** Tengo algunos problemas para caminar

**** Tengo que estar en la cama

Cuidado personal

**** No tengo problemas con el cuidado personal

**** Tengo algunos problemas para lavarme o vestirme

**** Soy incapaz de lavarme o vestirme

Actividades cotidianas (p. ej., trabajar, estudiar, hacer las tareas domésticas, actividades familiares o durante el tiempo libre)

**** No tengo problemas para realizar mis actividades cotidianas

**** Tengo algunos problemas para realizar mis actividades cotidianas

**** Soy incapaz de realizar mis actividades cotidianas

Dolor/malestar

**** No tengo dolor ni malestar

- Tengo moderado dolor o malestar
- Tengo mucho dolor o malestar

Ansiedad/depresión

**** No estoy ansioso ni deprimido

**** Estoy moderadamente ansioso o deprimido

**** Estoy muy ansioso o deprimido

Comparado con mi estado general de salud durante los últimos 12 meses, mi estado de salud hoy es:

**** Mejor

**** Igual

**** Peor

Para ayudar a la gente a describir lo bueno o malo que es su estado de salud hemos dibujado una escala parecida a un termómetro en la cual se marca con un 100 el mejor estado de salud que se pueda imaginar y con un 0 el peor estado de salud que se pueda imaginar. Nos gustaría que nos indicara en esta escala, en su opinión, lo bueno o

malo que es su estado de salud en el día de *hoy*.

Mejor estado de salud

Peor estado de salud

Su estado de salud hoy

**Apartado II**

**Escenario 1.**

Imaginemos por un momento que no existe el sistema público de salud. Suponga que usted tiene una necesidad de salud similar al que le ha traído hoy a la consulta y tiene que ser atendido por la misma enfermera que lo ha recibido hoy, pero tiene que pagar esa atención directamente.

Tarjeta 1¿Cuánto dinero estaría dispuesto a pagar por esta consulta?

** ** A: 0-20 euros

- B: 21-40 euros
- C: >40 euros

Tarjeta 2 ¿Cuánto dinero estaría dispuesto a pagar por esta consulta?

** ** A: 0 euros

- B: 5 euros
- C: 10euros
- D: 15euros
- E: 20euros

- F: 25euros
- G: 30euros
- H: 35euros
- I: 40euros
- J: 45euros
- K: 50euros
- L: 55euros
- M: 60euros
- N:>60euro**s**

**Si ha marcado la opción A se debe a:**

- No puedo permitirme pagar por este servicio
- No estoy dispuesto a pagar por este servicio
- No encuentro pertinente la pregunta
- Otras razones

**Si ha marcado la opción N, estaría dispuesto a pagar ___ euros**

**Escenario 2.**

En esta nueva situación imaginamos que usted tiene una necesidad de salud similar a la que le ha traído hoy a la consulta, pero el gobierno ha decidido que no se va a volver a prestar el servicio de esta manera, y que el ciudadano recibirá un cheque para compensar la pérdida por el servicio. ¿Cuál sería la mínima cantidad que exigiría recibir para no sentirse perjudicado por la pérdida de este servicio concreto?

Tarjeta 1

¿Cuál sería la mínima cantidad que exigiría recibir para no sentirse perjudicado por la pérdida de este servicio concreto?

** ** A: 0-20 euros

- B: 21-40 euros
- C: >40 euros

Tarjeta 2

¿Cuál sería la mínima cantidad que exigiría recibir para no sentirse perjudicado por la pérdida de este servicio concreto

** ** A: 0 euros

- B: 5 euros
- C: 10euros
- D: 15euros
- E: 20euros
- F: 25euros
- G: 30euros
- H: 35euros
- I: 40euros
- J: 45euros
- K: 50euros
- L: 55euros
- M: 60euros
- N:>60euros

**Si ha marcado la opción N, debería recibir al menos ____ euros**

**Apartado III**

**Respecto a la relación con su enfermera, se le presentan unas frases acerca de lo que una persona puede sentir acerca de ella. Escoja lo apropiado de cada frase con respecto a su caso marcando un número por frase (recuerde que lo que usted diga es confidencial y que nadie podrá acceder a sus contestaciones).**

**1.** Mi enfermera me escucha cuando le explico mis problemas de salud

| 1  En completo desacuerdo | 2  En desacuerdo | 3  No sabría decir | 4  De acuerdo | 5  En completo acuerdo |
| --- | --- | --- | --- | --- |

**2. Mi enfermera es amable, me trata con cortesía**

| 1  En completo desacuerdo | 2  En desacuerdo | 3  No sabría decir | 4  De acuerdo | 5  En completo acuerdo |
| --- | --- | --- | --- | --- |

**3. Confío en mi enfermera, creo en lo que me dice**

| 1  En completo desacuerdo | 2  En desacuerdo | 3  No sabría decir | 4  De acuerdo | 5  En completo acuerdo |
| --- | --- | --- | --- | --- |

**4. Estoy totalmente satisfecho/a de la visita con esta** enfermera

| 1  Nada apropiado | 2  Algo apropiado | 3  Apropiado | 4  Bastante apropiado | 5  Totalmente apropiado |
| --- | --- | --- | --- | --- |

**5. La** enfermera ha puesto mucha atención en examinar todos los problemas.

| 1  Nada apropiado | 2  Algo apropiado | 3  Apropiado | 4  Bastante apropiado | 5  Totalmente apropiado |
| --- | --- | --- | --- | --- |

**6. Seguiré los consejos de la** enfermera porque creo que son muy acertados.

| 1  Nada apropiado | 2  Algo apropiado | 3  Apropiado | 4  Bastante apropiado | 5  Totalmente apropiado |
| --- | --- | --- | --- | --- |

**7. Me he sentido cómodo/a hablando con la enfermera sobre temas muy** personales.

| 1  Nada apropiado | 2  Algo apropiado | 3  Apropiado | 4  Bastante apropiado | 5  Totalmente apropiado |
| --- | --- | --- | --- | --- |

**8. El tiempo que he pasado con la** enfermera ha sido algo corto.

| 1  Nada apropiado | 2  Algo apropiado | 3  Apropiado | 4  Bastante apropiado | 5  Totalmente apropiado |
| --- | --- | --- | --- | --- |

**9. La** enfermera me ha dado una información completa sobre mi tratamiento.

| 1  Nada apropiado | 2  Algo apropiado | 3  Apropiado | 4  Bastante apropiado | 5  Totalmente apropiado |
| --- | --- | --- | --- | --- |

**10. Algunos aspectos de la consulta con la enfermera podrían haber sido mejores.**

| 1  Nada apropiado | 2  Algo apropiado | 3  Apropiado | 4  Bastante apropiado | 5  Totalmente apropiado |
| --- | --- | --- | --- | --- |

**11. Hay algunas cosas que la e**nfermera no sabe de mí.

| 1  Nada apropiado | 2  Algo apropiado | 3  Apropiado | 4  Bastante apropiado | 5  Totalmente apropiado |
| --- | --- | --- | --- | --- |

**12. La enfermera ha escuchado con mucha atención todo lo que le he dicho.**

| 1  Nada apropiado | 2  Algo apropiado | 3  Apropiado | 4  Bastante apropiado | 5  Totalmente apropiado |
| --- | --- | --- | --- | --- |

**13. Pienso que la** enfermera me ha tratado de manera personalizada.

| 1  Nada apropiado | 2  Algo apropiado | 3  Apropiado | 4  Bastante apropiado | 5  Totalmente apropiado |
| --- | --- | --- | --- | --- |

**14. El tiempo que he estado con la enfermera no ha sido suficiente para comentarle**

todo lo que deseaba.

| 1  Nada apropiado | 2  Algo apropiado | 3  Apropiado | 4  Bastante apropiado | 5  Totalmente apropiado |
| --- | --- | --- | --- | --- |

**15. Después de la visita con la enfermera entiendo mucho mejor mi problema de s**alud.

| 1  Nada apropiado | 2  Algo apropiado | 3  Apropiado | 4  Bastante apropiado | 5  Totalmente apropiado |
| --- | --- | --- | --- | --- |

**16. La enfermera se ha interesado por mí no sólo a causa de mi enfermedad,** sino también como persona.

| 1  Nada apropiado | 2  Algo apropiado | 3  Apropiado | 4  Bastante apropiado | 5  Totalmente apropiado |
| --- | --- | --- | --- | --- |

**17. La** enfermera lo sabe todo sobre mí.

| 1  Nada apropiado | 2  Algo apropiado | 3  Apropiado | 4  Bastante apropiado | 5  Totalmente apropiado |
| --- | --- | --- | --- | --- |

**18. Creo que la** enfermera sabía realmente lo que yo estaba pensando.

| 1  Nada apropiado | 2  Algo apropiado | 3  Apropiado | 4  Bastante apropiado | 5  Totalmente apropiado |
| --- | --- | --- | --- | --- |

**19. Me hubiera gustado estar más tiempo con la** enfermera.

| 1  Nada apropiado | 2  Algo apropiado | 3  Apropiado | 4  Bastante apropiado | 5  Totalmente apropiado |
| --- | --- | --- | --- | --- |

**20. No estoy del todo satisfecho/a con la visita a la enfermera**.

| 1  Nada apropiado | 2  Algo apropiado | 3  Apropiado | 4  Bastante apropiado | 5  Totalmente apropiado |
| --- | --- | --- | --- | --- |

**21. Me resultaría difícil hablar con la** enfermera sobre temas personales.

| 1  Nada apropiado | 2  Algo apropiado | 3  Apropiado | 4  Bastante apropiado | 5  Totalmente apropiado |
| --- | --- | --- | --- | --- |

**Apartado IV**

**A continuación, nos gustaría hacerle algunas preguntas acerca de su aceptación o rechazo a las situaciones de riesgo.**

**¿Diría que usted es una persona que trata de evitar el riesgo o que es más arriesgada cuando hay algún posible beneficio? Señale por favor en esta escala de 1 a 10, cómo se considera usted de arriesgado/a**

**Totalmente contrario al riesgo =1**

**Totalmente propenso al riesgo = 10**

| **1** | **2** | **3** | **4** | **5** | **6** | **7** | **8** | **9** | **10** |
| --- | --- | --- | --- | --- | --- | --- | --- | --- | --- |

**Ahora imagine que usted puede tomar parte en un concurso en el que le van ofreciendo la posibilidad de elegir entre dos cajas o quedarse con una cantidad fija de dinero Una caja tiene dentro 200€ y la otra está vacía. Si decide concursar, su premio será el contenido de la caja que elija.**

**Señale sus preferencias en cada caso**

** Le ofrecen 40 euros – o - Concursar **

** Le ofrecen 70 euros – o - Concursar **

** Le ofrecen 100 euros – o - Concursar **

** Le ofrecen 130 euros – o - Concursar **

** No sabe / No responde**

**Imagine que ahora las reglas del concurso cambian. Le van ofreciendo dinero o puede concursar, pero si concursa debe pagar previamente 40 euros. El premio si concursa es el mismo que en el caso anterior, el contenido de la caja que escoja, sabiendo que una tiene 200 € y la otra nada.**

**Señale sus preferencias en cada caso**

** Le ofrecen 0 euros – o - Concursar **

** Le ofrecen 30 euros – o - Concursar **

** Le ofrecen 60 euros – o - Concursar **

** Le ofrecen 90 euros – o - Concursar **

** No sabe / No responde**

**Apartado V**

**Por último vamos a preguntarle por una serie de datos estadísticos que nos ayudarán a clasificar sus respuestas y a interpretar los resultados del estudio**

**El número de personas que viven en su domicilio es de __ personas (incluido/a usted)**

**De ellas tienen 16 o más años __ personas**

**Y tienen 15 o menos años __ personas**

**Su ocupación principal actualmente es de**

**** Ama/amo de casa.

**** Estudiante.

**** Trabajador.

**** Paro.

- Jubilado.

**El nivel superior de estudios que ha cursado hasta su finalización es**

**** No sé leer ni escribir.

**** Sin estudios.

**** Estudios primarios.

- Estudios secundarios (Bachiller, BUP, ESO, F.P.hasta 2º ciclo)
- Estudios superiores (diplomaturas, licenciaturas, y tercer ciclo)

**Mi último trabajo remunerado ha sido el de:**

**** I Gerentes, directivos, profesiones liberales.

**** II Cargos intermedios y gerentes comerciales.

**** III Trabajadores cualificados no manuales.

**** IVa Trabajadores cualificados manuales.

**** IVb Trabajadores manuales parcialmente cualificados.

**** V Trabajadores manuales no cualificados.

En caso de no realizar trabajo remunerado se considera la clase más alta dentro de la Unidad familiar. Personas en situación de paro o incapacidad temporal o permanente o jubiladas se clasificarán conforme al último trabajo ejercido

**Los ingresos mensuales de su Unidad familiar (sumando los de todos los familiares que viven en su domicilio) están comprendidos en el rango señalado con la letra:**

**** A: Menos de 600 euros

- B: 600- 1200 euros
- C: 1200-1800 euros
- D: 1800-2400 euros
- E: 2400- 3600 euros
- F: 3600- 4800 euros
- G. 4800-6000 euros
- H: 6000-7200 euros
- I: Más de 7200 euros
